# Effects of two-week machine massage on muscle properties in adolescent wrestlers

**DOI:** 10.3389/fphys.2023.1129836

**Published:** 2023-02-27

**Authors:** Guangcai Qu, Hongbo Wang, Guohai Zhou, Haiping Liu

**Affiliations:** Department of Sports Science, Wenzhou Medical University, Wenzhou, China

**Keywords:** massage, exercise-induced muscle fatigue, muscle stiffness, creatine kinase, wrestlers

## Abstract

**Objective:** This study aimed to investigate the effect of a two-week machine massage on the physical properties of the erector spinae and serum biochemical indexes of adolescent athletes after training.

**Methods:** Sixteen male adolescent wrestlers were recruited (age: 15 ± 1 year; height: 166 ± 7 cm; weight: 56 ± 7 kg) and randomly assigned to machine massage (MA, 8) and control (CO, 8) groups. Participants in the MA group received machine massage for 20 min after each wrestling training from Monday to Saturday (except on Thursday) for two weeks, while the participants in the CO group recovered naturally. Over the course of two weeks, all the participants underwent similar wrestling training program under the guidance of a professional coach. Before and after the intervention, serum urea and creatine kinase (CK) levels were measured in a fasting state. A Myoton Pro digital muscle evaluation system was used to measure the physical properties of the erector spinae, including the oscillation frequency, logarithmic decrement of a muscle’s natural oscillation, and dynamic stiffness.

**Results:** After two weeks of machine massage treatment, the dynamic stiffness of the erector spinae in the MA group decreased by 12.90% and that in the CO group increased by 2.34%, indicating a significant difference between the two groups (*p* = 0.04, *ƞ* ^2^ = 0.286). The decrease in the logarithmic decrement of a muscle’s natural oscillation value in the MA was significantly greater than that in the CO (*p* = 0.003, *ƞ* ^2^ = 0.286). Moreover, the serum CK values decreased by 33.84% in the MA group and by 1.49% in the CO group, despite a trend of change between the groups (*p* = 0.062, *ƞ2* = 0.084). No significant difference was found in the improvement in serum urea levels between the two groups after two weeks of treatment.

**Conclusion:** Results of the present study indicated that a two-week machine massage had a positive effect on the improvement of the physical properties of the erector spinae of wrestlers during training.

## 1 Introduction

Early sports specialization appears to be increasingly common among athletes (Jayanthi et al., 2013). In the nationwide competitive sports system in China, the organization of adolescent sports training is orderly and standardized, in which teenagers enter residential sports schools for sports specialization training and cultural learning by age. This kind of year-round engagement in intense training programs in a single sport at an early age may produce physiological adaptations, such as cardiovascular, respiratory, muscle, and bone (Zauner et al., 1989). Meanwhile, early sports specialization may result in injuries due to exercise-induced fatigue in some young athletes (DiFiori et al., 2014). As a result, the elimination of exercise-induced muscle fatigue in young athletes is a prerequisite to ensure sustained and effective completion of exercise load and prevention of sports injuries.

Massage is a widely used form of therapy, especially as an effective means for athletes to decrease muscle stiffness, increase joint range of motion, prevent delayed onset of muscle soreness, and improve exercise performance (Kang et al., 2021). Currently, many coaches and athletes believe in the beneficial effects of massage during training and competition. A study in the United Kingdom showed that for the past 11 years, massage treatment was administered for approximately 45% of the total time in physiotherapy treatment (Galloway and Watt, 2004). The mechanisms through which these goals are achieved are proposed to increase the range of motion (ROM), decrease muscle stiffness, increase blood flow and muscle temperature (Black et al., 2003), and reduce spinal reflex excitability (Morelli et al., 1999). Moreover, the mechanical pressure involved by massage decrease tissue adhesion, and increase muscle-tendon compliance by mobilizing and elongating shortened or adhered connective tissue, which results in a less stiff muscle-tendon unit (Magnusson S., 1998). Paradoxically, inconsistent findings have been conveyed to improve the physical properties of the muscles resulting from massage after exercise or training. A study reported that healthy women were massaged for 10 min after maximum intensity exercise on a bicycle ergometer, and the muscle stiffness was significantly reduced at 5 and 20 min after massage (Ogai et al., 2008). In healthy individuals, muscle stiffness decreased significantly after 7 min of massage on the gastrocnemius, and this effect was short-lived and quickly returned to baseline values after cessation of the massage (Eriksson Crommert et al., 2015). In contrast, no changes were found in passive calf muscle stiffness with or without a bout of 10-min deep tissue massage (Thomson et al., 2015). The inconsistent findings regarding the effects of massage on muscle physical properties after exercise may be caused by differences in massage techniques and duration, varying pressure, and degree of exercise-induced muscle fatigue (Weerapong et al., 2005; Janecki et al., 2011).

A reliable outcome measure of muscle properties is critical for assessing the therapeutic efficacy of massage after intervention. Currently, mature techniques for the diagnosis of muscle function, such as electromyography, magnetic resonance imaging, ultrasound imaging, and tensiomyography have been widely applied in the fields of clinical medicine and sports science (Goo et al., 2020). However, considering the complex testing process, high cost and inconvenience in the process of application, a hand-held, non-invasive myotonometry device (i.e., the Myoton Pro) has been applied in measuring muscle mechanical properties, which applies a brief (15 ms) mechanical impulse to elicit damped oscillations of the muscle, and then calculated the oscillation frequency (F), logarithmic decrement of a muscle’s natural oscillation (D), and dynamic stiffness of the muscle (S) (Li Ya-peng et al., 2018), which reflected muscle tension, elasticity, and stiffness, respectively. Logarithmic decrement of a muscle’s natural oscillation is inversely proportional to its elasticity. Myotonometers have been validated and demonstrated to be reliable in a variety of populations, including athletes (Pruyn et al., 2016), healthy individuals (Lam et al., 2015), and patients with chronic diseases (Chuang et al., 2012; Chuang et al., 2013).

Wrestling is a high-risk contact sport. Wrestlers usually perform flexion and extension and torsion at the waist, such as high bridge motion during training; however, trunk muscles are very important in supporting and stabilizing the lumbar spine (Iwai et al., 2004; Chaabene, H. et al., 2017). Wrestlers’ long-term training causes waist muscle fatigue, which leads to an increase in the probability of low back pain (Iwai et al., 2004). Additionally, chronic low back pain can be caused by trunk muscular weakness in young wrestlers (Carpenter and Nelson, 1999). A previous study reported that approximately 70% of wrestlers had low back pain (Lundin et al., 2001). Therefore, eliminating muscle fatigue in wrestlers after daily training is an important issue for improving training effect and quality. However, previous studies have focused on the effect of one-time massage, and few studies have investigated the cumulative effects of relatively long-term massage on exercise-induced fatigue (i.e., muscle stiffness).

This study aimed to investigate the effect of machine massage on physical properties and serum biochemical indices after a short-term habitual training in adolescent wrestlers. Given that the knowledge of exercise-induced symptoms of muscle soreness usually lasts for several days and that fatigue accumulation may lead to spontaneous muscle injuries in athletes. We hypothesized that relatively long-term machine massage (i.e., two weeks) would improve the physical properties of the muscle.

## 2 Materials and methods

### 2.1 Participants

This study was approved by the Ethics Committee of the Wenzhou Medical University (code: 2021–008). Written informed consent to participate in this study was provided by the participant’s legal guardian. A power calculation was performed to determine the sample size using G * Power (version 3.1) before recruitment. Assuming a large effect size of 0.64 based on serum CK levels and power of 0.80 with an alpha of 0.05, a total sample size of 16 participants was sufficient for the present study (Guo et al., 2017). Sixteen eligible male wrestlers from a residential sports school were included (age: 15 ± 1 year; height: 166 ± 7 cm; weight: 56 ± 7 kg), and all of them had more than three years of wrestling training experience, with no previous injuries during the six months prior to the study. Routine activities (exercise training, cultural learning, accommodation, and diet) were strictly scheduled.

### 2.2 Study design

Under the guidance of the same coach, all subjects underwent the same wrestling training program (attachment) in the week before the experiment as during the intervention, which included wrestling technique, strength, endurance, and flexibility exercises. The training load was evaluated by rating the perceived exertion (Borg 6–20 RPE scale).

The baseline muscle properties and serum biochemical indices were measured on Sunday morning, a day before the intervention. The participants were then randomly assigned to the massage group (MA, *n* = 8) or the control group (CO, *n* = 8) through lottery method.

**FIGURE 1:**
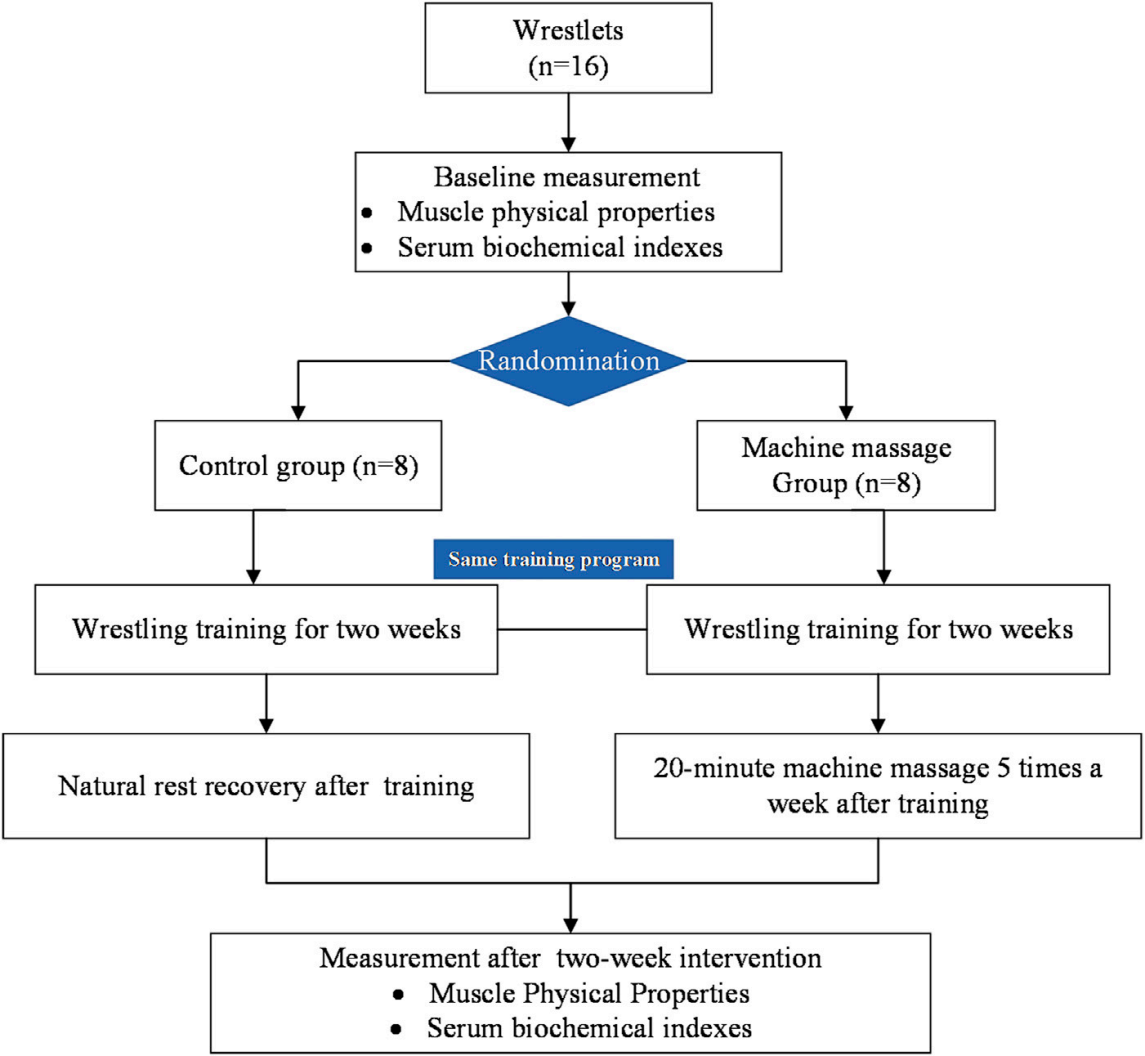
Flow-chart of the study.

Subsequently, the participants in the MA group received 20 min of machine massage after each wrestling training session on Mondays, Tuesdays, Wednesdays, Fridays, and Saturdays for two weeks, whereas the participants in the CO took a natural rest recovery for 20 min (Figure 1). No machine massage intervention was performed on Thursday because of the relatively small amount of training load on that day. Muscle properties and serum biochemical indices were measured again after the two-week machine massage intervention by the same investigator. During the study period, participants were requested to maintain a same diet and consistent routine activities (including morning cultural course learning, afternoon wrestling training and evening cultural course learning).

### 2.3 Machine massage schedule

**FIGURE 2:**
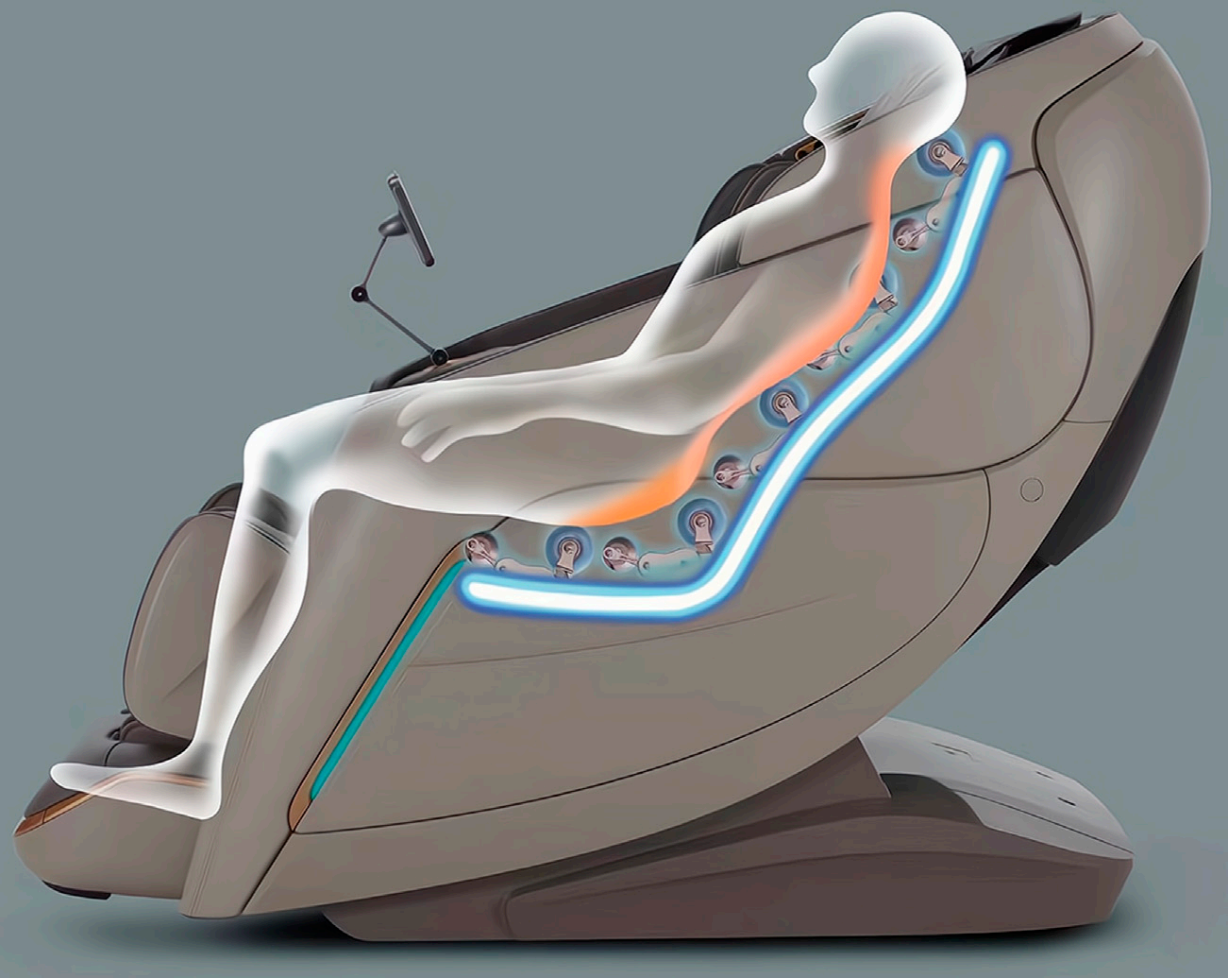
The position of mechanical arm of machine massage.

A massage chair (A307, iRest Health Technology Co. LTD., Zhejiang Province, P.R. China) was used as a massage technique for 20 min each time, five days a week for two weeks in the MA group. Thirty minutes after the wrestling training in the afternoon, the participants in the MA group leaned back in the massage chair in the resting room at a temperature of 25°C and humidity of 67%, whereas the mechanical arm kneaded the erector spinae muscle for 10 min first, then tapped for 10 min (Figure 2). The parameters of the massage chair included the kneading technique with a frequency of 1 Hz and an amplitude of 5–8 cm, and the tapping technique with a frequency of 10 Hz and an amplitude of 1–2 cm.

### 2.4 Parameters of muscle physical properties measurement

After obtaining blood samples, all the participants lay prostrate on a bed and were kept in a relaxed state. A hand-held myotonometry device, Myoton Pro (Myoton AS, Tallinn, Estonia), was used to measure oscillation frequency, logarithmic decrement of a muscle’s natural oscillation, and dynamic stiffness of the muscle. The erector spinae site was marked at spinal level L1, 1.5 cm away from the midline of the spine. The mean value of three consecutive measurements was used in the analysis. To maintain consistency before and after the experiment, measurement points were marked and photographed. The same tester took all the parameters of the muscle property measurements throughout.

### 2.5 Serum biochemical indexes measurement

The participants were instructed to refrain from caffeine 24 h before blood sampling and they arrived at the laboratory in the morning after a fasting state (12 h) prior to blood sampling. All the participants kept a quiet rest for 10 min in the laboratory (25°C and humidity 67%), and then 2 mL of blood sample was collected from the forearm vein in screw-cap polypropylene tubes (Sorfa, Zhejiang, China). Subsequently, serum was extracted at 5000 RPM for analysis (Martinez-Amat et al., 2005). Serum urea and creatine kinase levels were measured using the ultraviolet glutamate dehydrogenase method and International Federation Clinical Chemistry method, respectively, using an automatic biochemical analyzer (BS-220, Mindry, P.R. China). These measurements were performed in duplicate and the average of the two measurements was taken as the final result. The intra-assay coefficient of variation was both within 5.0%.

### 2.6 Statistical analysis

All data were analyzed using SPSS statistical software package (Release 25.0; IBM, New York, United States) and expressed as mean ± standard deviation. All variables showed a normal distribution according to the Shapiro-Wilk test. Descriptive statistics and independent sample t-tests were used for demographic and baseline variables. However, since the randomization method did not guarantee that the baseline characteristics would be similar between the groups, analyses of covariance were performed to compare the changes in the variables of the MA and CO groups after intervention. Baseline was used as the covariate to adjust the change values of the post-measurements. Considering the small sample size, effect size (ES) was used to assess the group effect, and the scores of partial *ƞ* ^*2*^ was considered small if ƞ^2^<0.01 and large if *ƞ* ^*2*^>0.14 (Kirk, 1996). Statistical significance was set at *p* < 0.05.

## 3 Results

### 3.1 Muscle physical properties of the erector spinae

**FIGURE 3:**
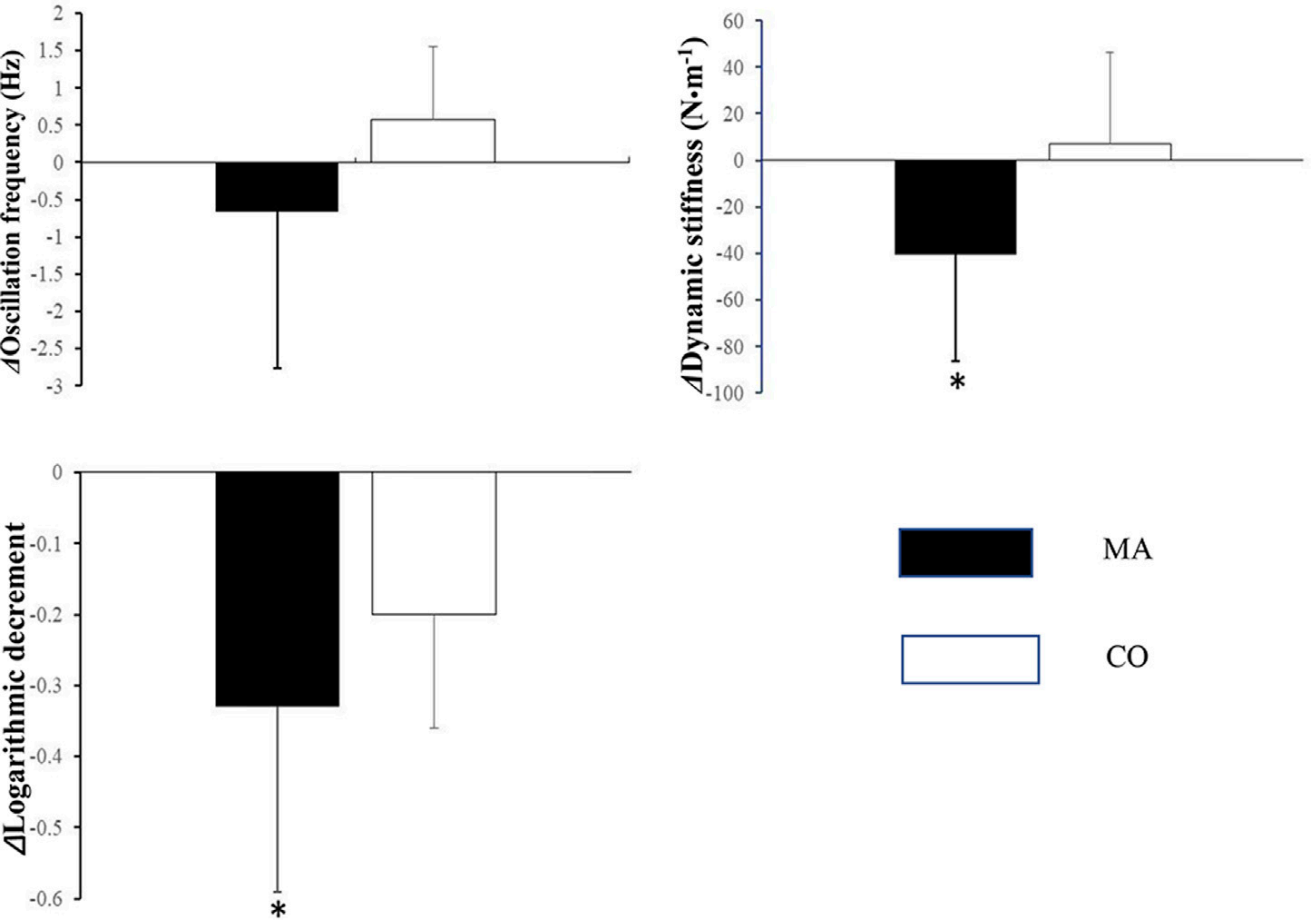
The change amplitude of physical properties of erector spine measured using Myoton Pro in MA and CO group after two-week intervention. (*: Significant difference compared with CO group, P<0.05).

Table 1 shows the influence of machine massage, lasting for two weeks on the physical properties of erector spinae the wrestlers after training. After two weeks of treatment, the oscillation frequency decreased by 3.79% in the MA group and increased by 3.41% in the CO group. There was only a trend changes in F between the two groups after the intervention (*F* = 3.690, *p* = 0.077, *ƞ* ^2^ = 0.221, Table 1; Figure 3).

**TABLE 1:** Influence of machine massage on muscle physical properties and serum biochemical indexes after intervention.

		CO					MA					Group effect	
		Pre		Post		Δ %	Pre		Post		Δ %	*p*	*Partial η* ^*2*^
Physical properties	Oscillation frequency (Hz)	17.00 ± 0.53		17.58 ± 1.07		+ 3.41	17.41 ± 2.31		16.75 ± 0.79		−3.79	0.077	0.221
	Dynamic stiffness (N∙m^-1^)	310.25 ± 12.44		317.50 ± 37.60		+ 2.34	315.88 ± 36.95		282.25 ± 23.74		−12.90	0.040	0.286
	Logarithmic decrement	1.13 ± 0.26		0.94 ± 0.15		−17.70	1.12 ± 0.18		0.81 ± 0.08		−29.46	0.007	0.437
Biochemical indexes													
	CK(U∙L)	514.28 ±265.57		506.63 ± 240.21		−1.49	729.74 ±244.27		482.81 ±180.07		−33.84	0.062	0.084
	Urea (mmol∙L)	5.21 ± 0.90		5.11 ± 1.20		−1.92	6.47 ± 1.28		5.77 ± 1.17		−10.82	0.304	0.088

Values are expressed as means ± standard deviation. CO: control group, MA: machine massage group. delta (Δ) for the change from before to after intervention. Partial value for effect size (ES). CK: creatine kinase. Logarithmic decrement: Logarithmic decrement of a muscle’s natural oscillation.

Dynamic stiffness in the MA group decreased by 12.90% and that in the CO group increased by 2.34% compared to before two weeks of machine massage treatment. A significant difference was found in the change in amplitude of dynamic stiffness of the erector spinae between the two groups (*F* = 5.219, *p* = 0.040, *ƞ* ^2^ = 0.286, Table 1; Figure 3).

After two weeks of machine massage treatment, the decrease in the logarithmic decrement of a muscle’s natural oscillation value was 29.46% and 17.70% in the MA and CO groups, respectively, and the former significantly reduced more than the latter (*F* = 10.106, *p* = 0.007, *ƞ* ^2^ = 0.437, Table 1; Figure 3).

### 3.2 Serum urea and CK

After two weeks of machine massage, serum CK levels decreased by 33.84% in the MA group and 1.49% in the CO group. Additionally, there was a trend change in serum CK (*p* = 0.062, *ƞ* ^2^ = 0.084) between the MA (246.93 ± 285.79 U/L) and CO (7.65 ± 172.64 U/L) groups. Nevertheless, no significant difference was found in the improvement in serum urea levels between the two groups after two weeks of treatment (Table 1).

## 4 Discussion

The results of this study showed that the dynamic stiffness and logarithmic decrement of a muscle’s natural oscillation of the erector spinae decreased by 13% and 30% in the MA group, and the dynamic stiffness increased by 3% and the logarithmic decrement of a muscle’s natural oscillation decreased by 18% in the CO group after two weeks of machine massage treatment, respectively. There was a significant difference in the change in amplitude of dynamic stiffness and logarithmic decrement of a muscle’s natural oscillation of the erector spinae between the two groups. These applied that two weeks of machine massage could reduce muscle stiffness and increase the muscle elasticity of the erector spinae of wrestlers, indicating that long-term machine massage could improve the physical characteristics of muscles after regular training in athletes.

A possible mechanism to explain the decrease in muscle stiffness during the following massage could be that pressure on the muscles during massage breaks the stable cross-bridges between the actin and myosin filaments (Proske and Morgan, 1999), and thus, increases intramuscular temperature, which decreases muscle shear elastic modulus (Sapin-de Brosses et al., 2010). Nevertheless, the beneficial effects of long-term massage on muscle physical properties may be the cumulative effects of each massage.

In recent years, there have been few studies on long-term massage to improve the physical properties of the muscles. Previous studies of one-time massage to reduce muscle stiffness have reported inconsistent results. Studies have shown that post-exercise recovery from measured muscle stiffness was more pronounced after massage than in control participants (Ogai et al., 2008). Massage after eccentric exercise had a greater effect on reducing muscle stiffness within a day rather than affecting recovery over several days in rabbits (Crawford et al., 2014). Similarly, enhancement of post-exercise relaxation of muscles by vibration treatment significantly lowered the values of post-exercise muscle stiffness in young men, indicating that vibration treatment seems to be a more effective method of restitution than passive resting (Chwała and Pogwizd, 2021). No changes were found in passive calf muscle stiffness with or without a bout of 10-min deep tissue massage (Thomson et al., 2015). Other experiments have also confirmed that post-exercise massage had no treatment effect on alleviating altered muscle stiffness in the major leg muscles of male recreational runners (Kong et al., 2018). Despite this, drawing conclusions from these studies should be done with caution, as there is a great amount of variance in the study methods and designs. For example, massage techniques varied, as did the time spent performing the different strokes. The overall consensus might be that massage reduces muscle stiffness; however, it remains unclear which technique and dosage are optimal. The experience of therapists was another inconsistency among studies (Nelson, 2013). Therefore, in this study, the massage chair was selected for massage, and the mechanical arm was massaged according to a pre-designed procedure to avoid the weakness of manual massage.

Creatine kinase and urea in the blood mainly originate from breakdown of muscle cells and synthesis in the liver, respectively. It has been documented that training affects creatine kinase activity and blood urea concentration. Therefore, the determination of serum creatine kinase activity and urea concentration after training could be used as effective biochemical indices to evaluate the fatigue state of athletes (Howatson and Van Someren, 2003), despite divergent views on the assessment of the blood levels of myofibre proteins (i.e., CK) based on the fact that the blood streams reflect not only their release into the blood but also their removal (Warren, et al., 1999). In this study, the young wrestlers received machine massage five times a week for 20 min each time for two weeks after training, and the serum CK level decreased by 33.83%. The improvement trend was better than that of the athletes who did not receive massage. Several studies have shown that massage and acupuncture can significantly decrease post-exercise creatine kinase and urea levels and promote muscle fatigue elimination in healthy volunteers (Zhou Yan and Yang Xiao-bo, 2003; Zainuddin et al., 2005). Tyka et al. (2018) also reported that vibro-massage could have a positive effect on the level of a marker of muscle damage and connective tissues after long-term physical exercise in males. The mechanisms underlying the effect of massage on creatine kinase levels remain unclear. Some possible reasons for the decrease in serum creatine kinase response for massage are lower creatine kinase efflux from the damaged muscle and increased clearance of creatine kinase from the circulation through increased blood and lymph flow (Zainuddin et al., 2005; Sefton et al., 2010). A previous study demonstrated that massage significantly elevated the temperature in massage areas, as well as in adjacent non-massage areas, which may lead to changes in peripheral blood flow (Sefton et al., 2010).

The present study has several limitations. First, changes in the physical properties of muscles were not tested after each massage, and it was impossible to investigate the trend of long-term massage on muscle characteristics. Second, the blood biochemical indices (serum urea and CK) were collected before and after the intervention; thus, the present study could not identify the extent of muscle damage and change process, resulting from the massage throughout the entire treatment period. Third, we did not include a placebo treatment group (e.g., touching), which does not exclude the psychological effect to some extent. Additionally, even though manual massage therapy has some disadvantages such as different operational techniques, time-consuming and even the challenges on the delivery of manual interventions to a large size patient, it has the benefits of complex maneuvers and personalized massage (Paul et al., 2021). Hence, it remains constraint whether manual massage can be substituted by its automated or robotic counterpart.”

## 5 Conclusion

The results of the present study suggest that the armchair massage technique could improve the physical properties of muscles after training of adolescent wrestlers and achieve similar positive effect as manual massage in improving muscle function. Monitoring muscle stiffness using myotonometry might provide useful information for coaches and clinicians regarding recovery status of athletes.

## Data Availability

The original contributions presented in the study are included in the article/Supplementary Material, further inquiries can be directed to the corresponding author.

## References

[B1] Black C. D. Vickerson B. McCully K. K. (2003). Noninvasive assessment of vascular function in the posterior tibial artery of healthy humans. Dyn. Med. 2, 1. 10.1186/1476-5918-2-1 12628021 PMC151670

[B2] Carpenter D. M. Nelson B. W. (1999). Low back strengthening for the prevention and treatment of low back pain. Med. Sci. Sports Exerc. 31, 18–24. 10.1097/00005768-199901000-00005 9927005

[B3] Chaabene H. Negra Y. Bouguezzi R. Mkaouer B. Franchini E. Julio U. (2017). Physical and physiological attributes of wrestlers: An update. J. Strength & Cond. Res. 31 (5), 1411–1442. 10.1519/JSC.0000000000001738 28030533

[B4] Chuang L. L. Lin K. C. Wu C. Y. Chang C. W. Chen H. C. Yin H. P. (2013). Relative and absolute reliabilities of the myotonometric measurements of hemiparetic arms in patients with stroke. Arch. Phys. Med. Rehabil. 94, 459–466. 10.1016/j.apmr.2012.08.212 22960277

[B5] Chuang L. L. Wu C. Y. Lin K. C. (2012). Reliability, validity, and responsiveness of myotonometric measurement of muscle tone, elasticity, and stiffness in patients with stroke. Arch. Phys. Med. Rehabil. 93, 532–540. 10.1016/j.apmr.2011.09.014 22222143

[B6] Chwała W. Pogwizd P. (2021). Effects of vibration and passive resting on muscle stiffness and restitution after submaximal exercise analyzed by elastography. Acta Bioeng. Biomech. 23, 3–11. 10.37190/ABB-01778-2020-02 34846035

[B7] Crawford S. K. Haas C. Butterfield T. A. Wang Q. Zhang X. Zhao Y. (2014). Effects of immediate vs. delayed massage-like loading on skeletal muscle viscoelastic properties following eccentric exercise. Clin. Biomech. (Bristol, Avon) 29, 671–678. 10.1016/j.clinbiomech.2014.04.007 24861827 PMC4112012

[B8] DiFiori J. P. Benjamin H. J. Brenner J. S. Gregory A. Jayanthi N. Landry G. L. (2014). Overuse injuries and burnout in youth sports: A position statement from the American medical society for sports medicine. Br. J. Sports Med. 48, 287–288. 10.1136/bjsports-2013-093299 24463910

[B9] Eriksson Crommert M. Lacourpaille L. Heales L. J. Tucker K. Hug F. (2015). Massage induces an immediate, albeit short-term, reduction in muscle stiffness. Scand. J. Med. Sci. Sports. 25, e490–e496. 10.1111/sms.12341 25487283

[B10] Galloway S. D. Watt J. M. (2004). Massage provision by physiotherapists at major athletics events between 1987 and 1998. Br. J. Sports Med. 38, 235–236. discussion 237. 10.1136/bjsm.2002.003145 15039270 PMC1724774

[B11] Goo M. Johnston L. M. Hug F. Tucker K. (2020). Systematic review of instrumented measures of skeletal muscle mechanical properties: Evidence for the application of shear wave elastography with children. Ultrasound Med. Biol. 46, 1831–1840. 10.1016/j.ultrasmedbio.2020.04.009 32423570

[B12] Guo J. Li L. Gong Y. Zhu R. Xu J. Zou J. (2017). Massage alleviates delayed onset muscle soreness after strenuous exercise: A systematic review and meta-analysis. Front. Physiol. 8, 747. 10.3389/fphys.2017.00747 29021762 PMC5623674

[B13] Howatson G. Van Someren K. A. (2003). Ice massage. Effects on exercise-induced muscle damage. J. Sports Med. Phys. Fit. 43, 500–505. pmid: 14767412. 14767412

[B14] Iwai K. Nakazato K. Irie K. Fujimoto H. Nakajima H. (2004). Trunk muscle strength and disability level of low back pain in collegiate wrestlers. Med. Sci. Sports Exerc. 36, 1296–1300. 10.1249/01.mss.0000135791.27929.c1 15292735

[B15] Janecki D. Jarocka E. Jaskólska A. Marusiak J. Jaskólski A. (2011). Muscle passive stiffness increases less after the second bout of eccentric exercise compared to the first bout. J. Sci. Med. Sport. 14, 338–343. 10.1016/j.jsams.2011.02.005 21414841

[B16] Jayanthi N. Pinkham C. Dugas L. Patrick B. Labella C. (2013). Sports specialization in young athletes: Evidence-based recommendations. Sports Health 5, 251–257. 10.1177/1941738112464626 24427397 PMC3658407

[B17] Kang L. Liu P. Peng A. Sun B. He Y. Huang Z. (2021). Application of traditional Chinese therapy in sports medicine. Sports Med. Health Sci. 3, 11–20. 10.1016/j.smhs.2021.02.006 35782678 PMC9219272

[B18] Kirk R. E. (1996). Practical significance: A concept whose time has come. Educ. Psychol. Meas. 56, 746–759. 10.1177/0013164496056005002

[B19] Kong P. W. Chua Y. H. Kawabata M. Burns S. F. Cai C. (2018). Effect of post-exercise massage on passive muscle stiffness measured using myotonometry - a double-blind study. J. Sports Sci. Med. 17, 599–606. pmid: 30479528. 30479528 PMC6243630

[B20] Lam W. K. Mok D. Lee W. C. C. Chen B. (2015). Reliability and asymmetry profiles of myotonometric measurements in healthy skeletal muscles. J. Nov. Physiother. 5, 245. 10.4172/2165-7025.1000245

[B21] Li Ya-peng F. Y. Zhu Yi Z. H. Liu Shu-fang Z. Z. (2018). Factors related to stiffness of gastrocnemius muscle: Study with Myoton PRO. Chin. J. Rehabil. Theor. Pract. 24, 442–446. 10.3969/j.issn.1006-9771.2018.04.012

[B22] Lundin O. Hellström M. Nilsson I. Swärd L. (2001). Back pain and radiological changes in the thoraco-lumbar spine of athletes. A long-term follow-up. Scand. J. Med. Sci. Sports. 11, 103–109. 10.1034/j.1600-0838.2001.011002103.x 11252458

[B23] Magnusson S. (1998). Passive properties of human skeletal muscle during stretch maneuvers. A review. Med. Sci. Sports Exerc 8, 65–77. 10.1111/j.1600-0838.1998.tb00171.x 9564710

[B24] Martinez-Amat A. Boulaiz H. Prados J. Marchal J. A. Puche P. P. Caba O. (2005). Release of α-actin into serum after skeletal muscle damage. Br. J. Sports Med. 39 (11), 830–834. 10.1136/bjsm.2004.017566 16244192 PMC1725075

[B25] Morelli M. Chapman C. E. Sullivan S. J. (1999). Do cutaneous receptors contribute to the changes in the amplitude of the H-reflex during massage? Electromyogr. Clin. Neurophysiol. 39, 441–447. 10.1016/S0009-2614(99)00203-1 10546081

[B26] Nelson N. (2013). Delayed onset muscle soreness: Is massage effective? J. Bodyw. Mov. Ther. 17, 475–482. 10.1016/j.jbmt.2013.03.002 24139006

[B27] Ogai R. Yamane M. Matsumoto T. Kosaka M. (2008). Effects of petrissage massage on fatigue and exercise performance following intensive cycle pedalling. Br. J. Sports Med. 42, 834–838. 10.1136/bjsm.2007.044396 18385196

[B28] Paul A. Usman J. Ahmad M. Y. Hamidreza M. Maryam H. Ong Z. C. (2021). Health efficacy of electrically operated automated massage on muscle properties, peripheral circulation, and physio-psychological variables: A narrative review. EURASIP J. Adv. Signal Process. 2021 (1), 80–22. 10.1186/s13634-021-00788-6

[B29] Proske U. Morgan D. L. (1999). Do cross-bridges contribute to the tension during stretch of passive muscle? J. Muscle Res. Cell Motil. 20, 433–442. 10.1023/a:1005573625675 10555062

[B30] Pruyn E. C. Watsford M. L. Murphy A. J. (2016). Validity and reliability of three methods of stiffness assessment. J. Sport Health Sci. 5, 476–483. 10.1016/j.jshs.2015.12.001 30356566 PMC6188913

[B31] Sapin-de Brosses E. Gennisson J. L. Pernot M. Fink M. Tanter M. (2010). Temperature dependence of the shear modulus of soft tissues assessed by ultrasound. Phys. Med. Biol. 55, 1701–1718. 10.1088/0031-9155/55/6/011 20197599

[B32] Sefton J. M. Yarar C. Berry J. W. Pascoe D. D. (2010). Therapeutic massage of the neck and shoulders produces changes in peripheral blood flow when assessed with dynamic infrared thermography. J. Altern. Complement. Med. 16, 723–732. 10.1089/acm.2009.0441 20590481

[B33] Thomson D. Gupta A. Arundell J. Crosbie J. (2015). Deep soft-tissue massage applied to healthy calf muscle has no effect on passive mechanical properties: A randomized, single-blind, cross-over study. BMC Sports Sci. Med. Rehabil. 7, 21. 10.1186/s13102-015-0015-8 26396740 PMC4578668

[B34] Tyka A. K. Palka T. Piotrowska A. Žižka D. Pilch W. Cebula A. (2018). The effect of vibro-massage on the level of selected markers of muscle damage and connective tissues after long-term physical exercise in males. J. Kinesiol. Exer. Sci. JKES. 82, 21–27. 10.5604/01.3001.0013.5088

[B35] Warren G. L. Lowe D. A. Armstrong R. B. (1999). Measurement tools used in the study of eccentric contraction-induced injury. Sports Med. 27 (1), 43–59. 10.2165/00007256-199927010-00004 10028132

[B36] Weerapong P. Hume P. A. Kolt G. S. (2005). The mechanisms of massage and effects on performance, muscle recovery and injury prevention. Sports Med. 35, 235–256. 10.2165/00007256-200535030-00004 15730338

[B37] Zainuddin Z. Newton M. Sacco P. Nosaka K. (2005). Effects of massage on delayed-onset muscle soreness, swelling, and recovery of muscle function. J. Athl. Train. 40, 174–180. 10.1152/japplphysiol.00163.2005 16284637 PMC1250256

[B38] Zauner C. W. Maksud M. G. Melichna J. (1989). Physiological considerations in training young athletes. Sports Med. 8, 15–31. 10.2165/00007256-198908010-00003 2675252

[B39] Zhou Yan W. Y. Yang Xiao-bo Z. L. (2003). Effect of acupuncture point stimulus on blood urea of sprinters after intensive speed endurance training. J. Shanghai Phys. Educ. Inst. 27, 74–77. 10.16099/j.cnki.jsus.2003.01.019

